# Proteomic analysis of intracellular protein corona of nanoparticles elucidates nano-trafficking network and nano-bio interactions

**DOI:** 10.7150/thno.38900

**Published:** 2020-01-01

**Authors:** Mengmeng Qin, Jian Zhang, Minghui Li, Dan Yang, Dechun Liu, Siyang Song, Jijun Fu, Hua Zhang, Wenbing Dai, Xueqing Wang, Yiguang Wang, Bing He, Qiang Zhang

**Affiliations:** 1 Beijing Key Laboratory of Molecular Pharmaceutics and New Drug Delivery Systems, School of Pharmaceutical Sciences, Peking University, Beijing, 100191, China.; 2 State Key Laboratory of Natural and Biomimetic Drugs, Peking University, Beijing, 100191, China.; 3 School of Food and Biological Engineering, Shaanxi University of Science and Technology, Xi'an, 710021, China.; 4 School of Pharmaceutical Science, Guangzhou Medical University, Guangzhou, 511436, China.

**Keywords:** intracellular protein corona, quantitative proteomics, nanoparticles, transcytosis, nano-bio interaction.

## Abstract

The merits of nanomedicines are significantly impacted by the surrounding biological environment. Similar to the protein corona generated on the surface of nanoparticles in the circulation system, the intracellular protein corona (IPC) might be formed on nanoparticles when transported inside the cells. However, little is known currently about the formation of IPC and its possible biological influence.

**Methods**: Caco-2 cells, a classical epithelial cell line, were cultured in Transwell plates to form a monolayer. Gold nanoparticles (AuNPs) were prepared as the model nanomedicine due to their excellent stability. Here we focused on identifying IPC formed on the surface of AuNPs during cell transport. The nanoparticles in the basolateral side of the Caco-2 monolayer were collected and analyzed by multiple techniques to verify IPC formation. High-performance liquid chromatography-tandem mass spectrometry (LC-MS/MS)-based proteomics was utilized to analyze the composition of IPC proteins. In particular, we established a dual-filtration strategy to exclude various interference in IPC identification. Based on the subcellular localization of specific IPC proteins, we elicited the nano-trafficking network of AuNPs. The transport pathways of AuNPs identified by proteomic analysis were also verified by various conventional technologies. Finally, we explored the influence of IPC on the uptake and stress response of endothelium.

**Results**: The existence of IPC was demonstrated on the surface of AuNPs, in which 227 proteins were identified. Among them, 40 proteins were finally ascertained as the specific IPC proteins. The subcellular location analysis indicated that these “specific” IPC proteins could back-track the transport pathways of nanoparticles in the epithelial cell monolayer. According to the subcellular distribution of IPC proteins and co-localization, we discovered a new pathway of nanoparticles from endosomes to secretory vesicles which was dominant during the transcytosis. After employing conventional imageology and pharmacology strategies to verify the result of proteomic analysis, we mapped a comprehensive intracellular transport network. Our study also revealed the merits of IPC analysis, which could readily elucidate the molecular mechanisms of transcytosis. Besides, the IPC proteins increased the uptake and stress response of endothelium, which was likely mediated by extracellular matrix and mitochondrion-related IPC proteins.

**Conclusion**: The comprehensive proteomic analysis of IPC enabled tracing of transport pathways in epithelial cells as well as revealing the biological impact of nanoparticles on endothelium.

## Introduction

Over the last decades, nanotechnology is increasingly changing our understanding of biomedical science, especially theranostics 1-3. The availability of multiple nanomedicines for diagnosis and therapy has significantly improved the treatment efficacies compared to the traditional modalities 4, 5. Nanomaterials can be functionally modified with different probes and ligands and therefore have obvious merits for the targeted delivery 6, 7. By accumulating in the targeted site, nanomaterials increase local concentrations of drugs and/or enhance the signal intensities for diagnosis. Nevertheless, due to the unclear biological behaviors, possible toxicities, and the availability of limited information in this regard, the clinical application of nanodrugs is limited despite their substantial biomedical potential as demonstrated by lots of fundamental studies 8, 9. Thus, exploring the nano-bio interactions and clarifying the biological effects are crucial for the clinical development of nanomedicines.

Independent of the administration route, nanomedicines are exposed to different biological milieus once they enter the body 10. It means that the nano-bio interactions exist in the whole process of drug absorption and distribution. Recently, it has been reported that, upon exposure to the blood circulation, nanoparticles adsorb many plasma proteins on their surface, forming a new interface termed “protein corona (PC)” 11. It is now widely acknowledged that PC mediates interactions between nanoparticles and biological environment 12. Since PC is what cells really face instead of nanoparticles themselves 13, the existence of PC can alter the biological behavior and functions of nanomedicines 14, 15. The analysis of PC would certainly further our understanding of the nano-bio interaction.

The blood circulatory system is not the only physiological environment that the nanomedicines encounter upon delivery 16, 17. In fact, nanomaterials contend with diverse cell milieus, especially epithelial cells, including gastrointestinal, intratracheal, intranasal, vaginal, and intrauterine cells, which constitute the primary barriers for drug absorption and distribution in the body 18, 19. Nanomedicines have to be transported across the epithelial monolayers before arriving at the targeting tissue 20. Generally, transcytosis is the primary mechanism for the transport across the cell monolayer, by which nanomedicines are internalized by cells from one side and transferred out of the other side 21. It is conceivable that intracellular proteins may interact with nanoparticles during the cellular transportation process. During the transport of nanoparticles through the epithelium, endogenous proteins may adsorb onto the particle surface and form the “intracellular protein corona (IPC)”. In analogy to the plasma PC, these proteins may mediate the nano-bio interactions and even change the therapeutic efficacy of nanomedicines. However, compared to numerous studies on plasma PC, not much information is available on the identification and analysis of IPC.

The IPC analysis may provide an extra function that differs entirely from the investigations of plasma PC. It has been reported that PC can record the places where the nanoparticles have passed 22-24. When nanomedicines are transported through various endomembrane organelles during transcytosis, specific proteins in these organelles may adsorb on particle surface and transfer out of cells as components of IPC. We hypothesized that these specific proteins in IPC might help in back-tracking the transport pathways of nanomedicines in cells. In other words, based on the organelle locations of these specific proteins, the nano-trafficking routes can be clarified. Although there are a few of studies related to this issue already 25, 26. However, the present studies failed to address how to back-track the transcytosis pathways of nanoparticles by proteomic methods. Moreover, we still do not how IPC formed during intracellular trafficking affects the subsequent transportation of nanomedicines.

In this study, we established a mass spectrometry (MS)-based proteomic strategy to detect the composition of IPC after transcytosis, and explore the functions of IPC proteins to trace the transport pathways as well as reveal the biological impact of nanoparticles. Gold nanoparticles (AuNPs) were fabricated as the model nanomedicine because of their excellent stability with respect to both physicochemical properties and morphology 27 and are extensively utilized for tumor chemotherapy, photothermal therapy and radiotherapy 28-30. Caco-2 cells were used as the cell monolayer model for transcytosis detection 31, 32. The formation of IPC was verified by transmission electron microscopy (TEM) and gel electrophoresis analysis. Notably, we established a dual-filtration strategy based on LC-MS/MS to analyze the organelle-specific proteins in IPC. Based on the information obtained from LC-MS/MS, we remodeled the cellular transport network of AuNPs at a molecule level during transcytosis. Exocytosis, an adverse nano-trafficking route that differs from the transcytosis, was also systematically investigated in this study. Thus, the influence of transport direction on IPC composition could be clarified. Finally, we detected the uptake of AuNPs by endothelial cells after transcytosis, which reflected the biological effects of IPC on the nano-bio interactions.

## Results and Discussion

### Gold nanoparticles were fabricated with good dispersity and stability

**Figure 1:**
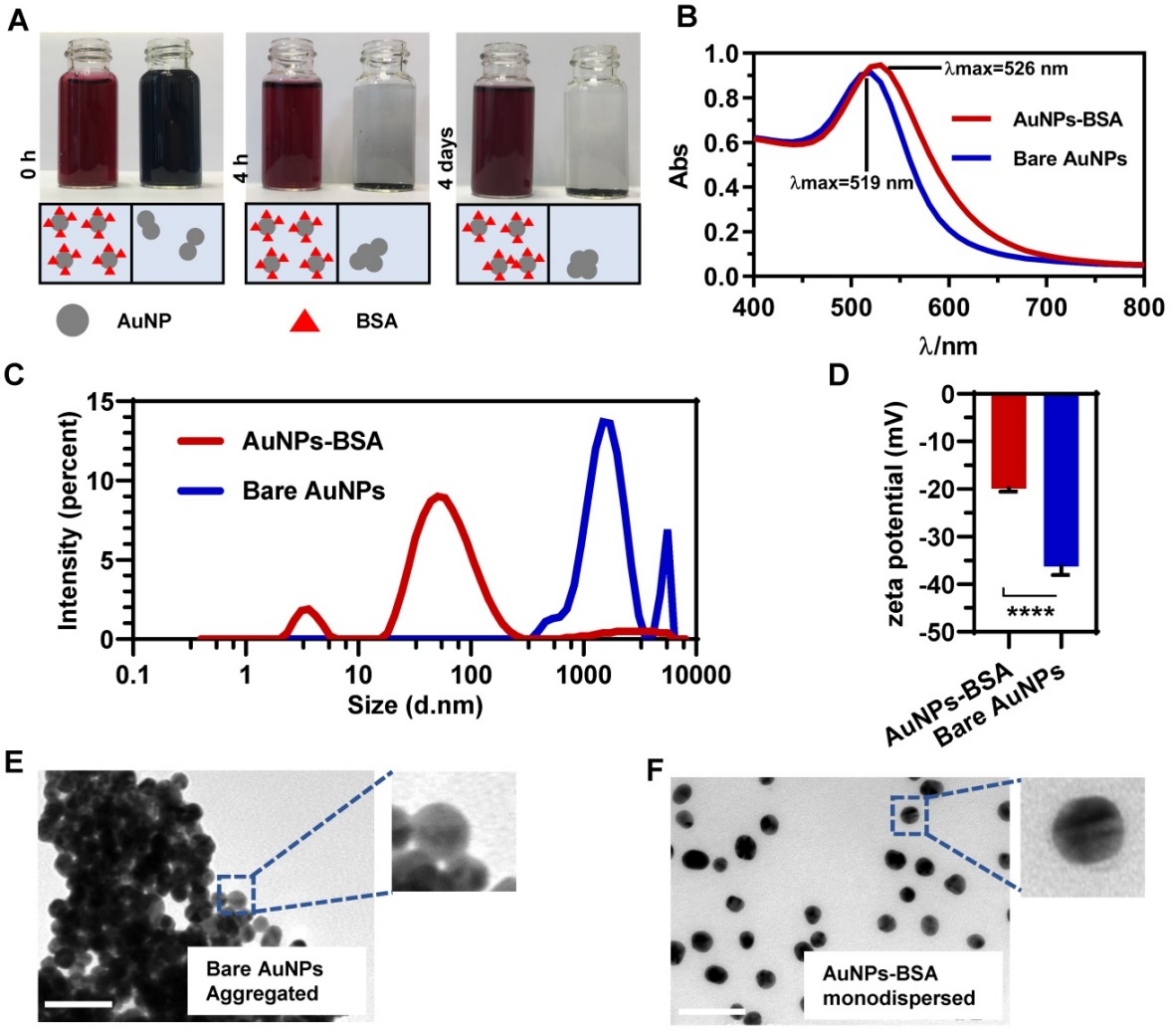
** Characterization of gold nanoparticles.** (A) Appearance of Bare AuNPs and AuNPs-BSA mixed with an equal volume of phenol red and serum-free DMEM after 0 h, 4 h or 4 days. (B) UV-vis absorbance spectra of Bare AuNPs and AuNPs-BSA dispersed in water. (C) Distribution of the hydrodynamic diameter of Bare AuNPs and AuNPs-BSA dispersed in serum-free DMEM. (D) Zeta potential of Bare AuNPs and AuNPs-BSA dispersed in water. Mean ± SD, n = 6, ****p < 0.0001. (E) Morphologies of Bare AuNPs dispersed in serum-free DMEM under TEM. (F) Morphologies of AuNPs-BSA dispersed in serum-free DMEM under TEM. Scale bar TEM, 50 nm.

**Figure 2:**
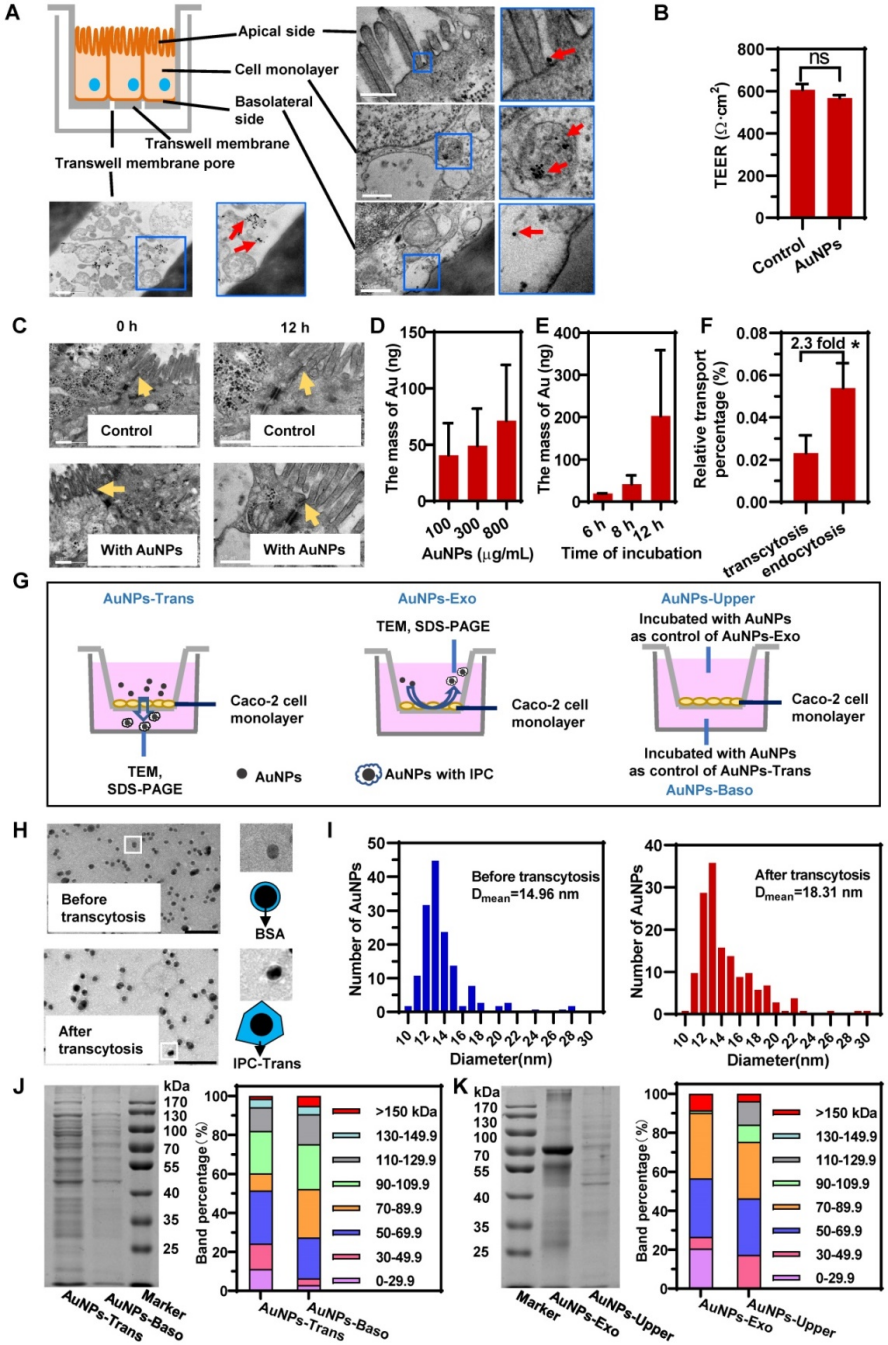
** Intracellular proteins adsorbed on the surface of AuNPs to form IPC after transcytosis and exocytosis.** (A) Schematic diagram of the Caco-2 monolayer on Transwell and distribution of AuNPs in different parts of Caco-2 monolayer during transcytosis. Red arrows indicate the AuNPs. Scale bar TEM, 500 nm. (B) TEER of Caco-2 monolayer before and after incubation with 800 μg/mL AuNPs for 12 h. (C) Morphology of tight junctions of Caco-2 monolayer incubated with or without 800 μg/mL AuNPs for 12 h. Yellow arrows indicate the tight junctions. Scale bar TEM, 500 nm. (D) Effect of concentration of AuNPs on transcytosis. Caco-2 cell monolayer was incubated with different concentration of AuNPs for 8 h. (E) Effect of incubation time with AuNPs on transcytosis. Caco-2 cell monolayer was incubated with 500 μg/mL AuNPs for different time. (F) Relative transport ratio of AuNPs on Caco-2 monolayer for transcytosis and endocytosis. The percentages represent the ratio of transcytosis or endocytosis of nanoparticles to the total amount of AuNPs added. Mean ± SD, n = 3, *p < 0.05. (G) Schematics illustrate the differences among four groups of AuNPs. AuNPs-Trans refers to AuNPs collected from the basilar compartment of Transwell with Caco-2 monolayer after incubation with 800 μg/mL AuNPs for 12 h. AuNPs-Exo refers to AuNPs collected from the upper compartment of Transwell with Caco-2 monolayer after incubation with AuNPs for 12 h. AuNPs blended with liquid acquired from basilar and upper compartment of Transwells with Caco-2 monolayer separately were AuNPs-Baso and AuNPs-Upper. They were used as controls of AuNPs-Trans and AuNPs-Exo, respectively. (H) Morphology of AuNPs before and after transcytosis captured by negatively stained TEM. Scale bar TEM, 100 nm. (I) Diameter distribution of AuNPs before and after transcytosis. The diameter of AuNPs was measured by Image Pro Plus software (IPP) according to the TEM photos. n > 250. (J, K) SDS-PAGE of the proteins adsorbed on nanoparticles (left). Molecular weight distribution of adsorbed proteins was analyzed using the Bio-Rad software by calculating the gel band intensity on SDS- PAGE (right).

AuNPs were synthesized by reducing hydrogen tetrachloroaurate with sodium citrate (Figure S1). To maintain the dispersive stability of AuNPs in physiological environment, the prepared bare nanoparticles (Bare AuNPs) were incubated with a small amount of bovine serum albumin (BSA). Figure 1A shows that BSA-coated AuNPs (AuNPs-BSA) exhibited the crimson appearance compared to the black Bare AuNPs upon dispersion in serum-free DMEM. BSA molecules reduced the interparticle interactions and increased the dispersity of AuNPs. The UV-Vis absorbance spectrum in Figure 1B shows that the maximum absorption of AuNPs slightly red-shifted from 519 nm to 526 nm after incubation with BSA. In terms of the stability, AuNPs-BSA retained the consistent color after long-time incubations, but Bare AuNPs precipitated within a short time (Figure 1A). This difference was also verified by the dynamic light scattering (DLS) technology. As shown in Figure 1C, most AuNPs-BSA exhibited the average hydrodynamic diameter of near 30 nm in the culture medium. The lower peak of about 5 nm in the size distribution might be derived from the free BSA molecules. On the contrary, Bare AuNPs aggregated to bigger particles in micrometer size due to the interparticle affinity. Besides, the zeta potential of AuNPs altered from -36.2 mV to -19.8 mV following the BSA coating (Figure 1D), an observation that was consistent with the previous studies 33. TEM was utilized to monitor the morphological characteristics of AuNPs in the medium directly. Compared to the pristine AuNPs displayed in Figure 1E, BSA significantly blocked the particle coagulation (Figure 1F). The size distribution based on the TEM detection also exhibited the homogeneity and mono-dispersity of AuNPs-BSA (Figure 2I). Besides, AuNPs-BSA displayed no cytotoxicity in Caco-2 cells at the concentration used in the experiment (Figure S2). We also measured the protein concentration using Bradford assay and found that only 9.86 ng BSA adsorbed on particle surface for each microgram of AuNPs. In summary, we constructed a well-dispersed system of BSA-coated AuNPs, laying the foundation for the subsequent cellular transport studies.

### Intracellular proteins adsorbed on the surface of AuNPs to form IPC after transcytosis and exocytosis

As displayed in the schematic in Figure 2A**,** we first obtained an epithelial monolayer by culturing Caco-2 cells on Transwell membranes. AuNPs were incubated on the apical side for the transcytosis study. The detection of trans-epithelial electric resistance (TEER) indicated that the incubation of AuNPs did not affect cellular integrity (Figure 2B). The same result was also confirmed by confocal laser scanning microscopy (CLSM) (Figure S3). Besides, the TEM investigation illustrated that the tight junctions of cell monolayer were not perturbed by AuNPs (Figure 2C). These data suggested that AuNPs could not transfer across the epithelium by paracytosis. As shown in the TEM images displayed in Figure 2A, AuNPs bound to the apical membrane, triggered endocytosis, transferred to the basolateral side, and finally got out of the cells. The transcytosis process was also concentration- and time-dependent (Figure 2D-E). However, the quantitative detection shown in Figure 2F indicated that transcytosis of AuNPs was very low relative to the incubation amount, and was also lower than the uptake by the cell monolayer. These results suggested that the transcytosis efficiency of nanoparticles was low.

Next, we investigated the potential nano-protein interactions during cellular transport. It is worth mentioning here that due to epithelial cell polarity, the nano-trafficking out of cells might be with a different directionality. AuNPs could be transported from apical to basolateral sides (A-B) via transcytosis; they could also be internalized through apical membrane and also transported out from the apical side (A-A) by exocytosis. Compared to AuNPs-BSA before incubation, AuNPs after the transcytosis and exocytosis exhibited thicker and more irregular corona-like structures detected by TEM (Figure 2H, Figure S4). The comparison of size distribution also demonstrated a thicker protein coating for post-transcytosis AuNPs (Figure 2I). These findings indicated that intracellular proteins might coat the particle surface to form protein corona during transcytosis and exocytosis.

The intracellular protein coronas on AuNPs after transcytosis (AuNPs-Trans) and AuNPs after exocytosis (AuNPs-Exo) were further confirmed using SDS-PAGE. The comparison between AuNPs-Trans or AuNPs-Exo and their respective control groups could accurately reflect the IPC proteins by eliminating the interference of secreted proteins. Because the control group consisted of AuNPs-BSA incubated with the mediums acquired from the cell monolayer cultured without AuNPs and the proteins in the medium were mostly secreted proteins. As shown in Figure 2J and 2K, compared to the control groups, more proteins were detected on the surface of AuNPs after transcytosis and exocytosis, demonstrating the formation of the intracellular protein corona. Also, it illustrated the significant difference of IPC components between Trans and Exo groups, indicating that different transport directions triggered different nano-protein interactions. Interestingly, molecular weight (MW) distributions in Figure 2J and 2K revealed that low MW (LMW, <29.9 kDa) proteins in IPC constituted a higher proportion than in the control groups. Although the detailed composition remained to be determined, LMW proteins might be specific for the nano-bio interactions.

### Intracellular protein corona was identified by proteomics

We identified the corona compositions derived from different types of nano-protein interactions. Besides the IPC proteins that interacted with AuNPs during the intracellular transport, the extracellular secretory proteins and cell debris might also bind with nanoparticles when they transported out from the epithelium. To eliminate such interference, we performed two reference investigations via LFQ proteomics. First, fresh culture medium was incubated with the cell monolayer for a specific time. The basolateral medium was then collected and mixed with AuNPs to induce the formation of protein corona (PC) that we termed as “PC-Baso”, which was mainly composed of the secretory proteins. Second, we prepared the whole cell lysate and incubated with AuNPs and the resultant corona, named as “PC-Lysate”, which reflected the cell debris. We compared the IPC data after transcytosis (IPC-Trans) with PC-Baso or PC-Lysate groups and used a dual-filtration method to exclude the pseudo-IPC proteins. Thus, authentic intracellular nano-bio interactions could be clarified. Likewise, the same strategy was utilized for the IPC analysis after the exocytosis of AuNPs.

For each proteomic investigation, two independent biological replicates were conducted and the correlation coefficient (C_corr_) was calculated to evaluate the reproducibility. The data scatter diagrams in Figure S5 showed that all C_corr_ values exceeded 0.8, indicating the LFQ proteomics was feasible for the protein corona analysis.

**Figure 3:**
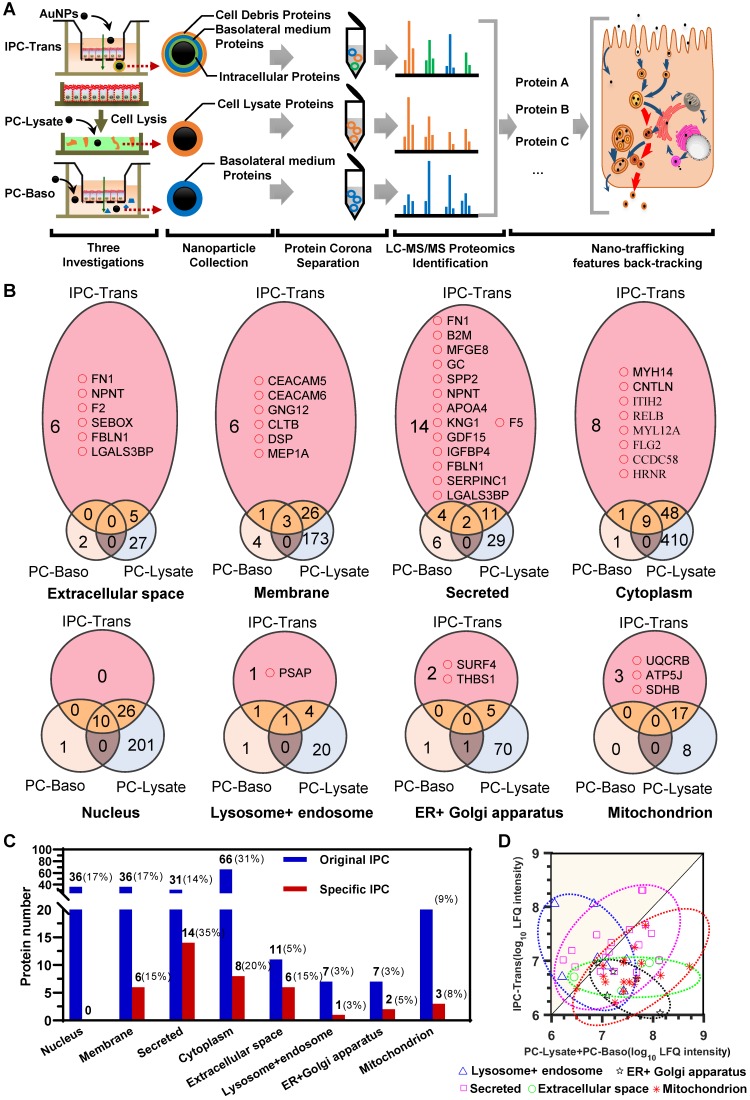
** The IPC proteins was identified by dual-filtration proteomic analysis.** (A) Flowchart of proteomic investigation for identification and analysis of the protein composition in IPC after transcytosis. (B) Venn diagrams of identified proteins among different PC groups. The pink area indicates the specific IPC and the yellow area refers to the shared IPC proteins of IPC-Trans over PC-Baso or PC-Lysate. (C) Protein numbers of original IPC and specific IPC located in different subcellular components. (D) Scatter diagram of shared IPC proteins. The data point above the diagonal line indicates the higher surface adsorption in the IPC-Trans group compared to the other two reference groups for the same protein.

For the IPC-Trans group, 227 proteins were identified in the original protein corona after the transcytosis of AuNPs (Figure S6). Due to the interference of extracellular proteins as described above, the original distribution of IPC components could not reflect the real context of AuNPs in cells. We, therefore, identified the proteins in PC-Baso and PC-Lysate groups, and found 49 and 781 proteins, respectively (Supplementary Data). The Venn diagrams among three groups in Figure 3B and Figure S7 show the specific IPC proteins (pink color field) in different cellular component (CC) classifications. Notably, as shown in Figure 3B-C, the proportion of specific IPC proteins based on CC was different from the original distribution in the IPC-Trans group.

### Analysis of IPC back-tracked nano-trafficking features in the epithelium

For the IPC analysis, the gene ontology (GO) based on CC further illustrated that near one third of proteins (29.1%) belonged to the cytoplasm, 15.9% were membrane-related proteins, and approximately 16% derived from the nucleus (Figure S6). According to Figure 3B-C, no nucleoprotein was detected in the transport of AuNPs, indicating that the nucleus was not involved. The majority of specific IPC proteins derived from the membrane component, secretory pathways, and extracellular space. The analysis indicated that most AuNPs might transfer across the epithelium through the vesicle-mediated secretory pathway. In this scenario, AuNPs were internalized into the secretory vesicles interacting with membrane or proteins that were about to be secreted to extracellular space or located in membrane. Some cytoplasmic proteins still bound with AuNPs during transport, suggesting that nanoparticles might penetrate through the cell membrane and enter the cytoplasm.

We also investigated the distribution of specific IPC proteins in subcellular organelles according to the pathway classification of nano-trafficking in cells. As shown in Figure 3B, several organelle proteins specifically interacted with AuNPs. A precursor protein of saposin, prosaposin (PSAP), which participates in the hydrolysis of sphingolipids, was identified. Reportedly, PSAP was transported via the ligand-mediated endocytosis and distributed throughout the whole pathway from endosomes to lysosomes 34, suggesting that AuNPs also underwent this route. In terms of the endoplasmic reticulum (ER)/Golgi apparatus, isoform 3 of the Surfeit locus protein 4 (SURF4) was discovered. It was located in the ER-Golgi intermediate compartment (ERGIC), maintaining the architecture of ERGIC and regulating the cargo transportation between the ER and Golgi apparatus 35. This finding directly demonstrated the involvement of the ER/Golgi apparatus during transcytosis. Thrombospondin-1 (THBS1), partially located on ER 36, was also detected, further showing the involvement of the ER/Golgi pathway to some extent. Furthermore, three specific proteins, ubiquinol-cytochrome c reductase complex (UQCRB), ATP synthase-coupling factor 6 (ATP5J) and succinate dehydrogenase iron-sulfur subunit (SDHB) 37-39, were detected confirming mitochondria to be another transfer site. In summary, the dual-filtration analysis of these transport pathways demonstrated that mitochondria, endosomes/lysosomes, and ER/Golgi apparatus were all involved in the transcytosis of AuNPs.

We subsequently analyzed the shared proteins among the three groups. Although these proteins were not the specific markers for the transport evaluation, their relative ratios could be used for the nano-trafficking characterization. We detected and compared the intensity ratios of shared proteins in three groups. As shown in the scatter diagram in **Figure 3D**, the data point above the diagonal line indicated the higher surface adsorption in the IPC-Trans group compared to the other two reference groups for the same protein. In general, the adsorption amount of proteins on the particle surface increased with the extension of incubation time 40. It indicated that AuNPs had interacted more strongly with these “upper line” proteins in cells before they were transported out of the epithelium. In other words, the intracellular organelle where these proteins were located was the main transport site for nano-trafficking. By classifying all data in **Figure 3D** based on the CC in GO, we found that the “upper line” proteins belonged to only two subcellular classifications, endosomes/lysosomes and secretory systems, indicating that these were the primary transfer stations during the transcytosis of nanoparticles.

As shown in Figure S7, the Venn diagrams exhibited specific IPC proteins after exocytosis of AuNPs (IPC-Exo). Notably, no specific protein from the nucleus, mitochondrion, ER/Golgi apparatus and extracellular space was detected, indicating that all these subcellular structures played little role in exocytosis. Similar to the IPC-Trans group, both endosomes/lysosomes and secretory vesicles participated in the nano-trafficking, since the corresponding marker proteins and membrane proteins were detected (Figure S8). Several cytoplasmic proteins were also found in IPC-Exo group, suggesting that a fraction of AuNPs might transfer out of cells through direct penetration mechanism.

In summary, we accurately identified and screened the specific IPC proteins after the transcytosis and exocytosis of nanoparticles using proteomic profiling based on our dual-filtration strategy. By analyzing the subcellular locations of these proteins and comparing the protein numbers among different cellular components, the nano-trafficking features in the epithelium could be back-tracked. With respect to transcytosis, the core transfer site was the secretory vesicles, where the nanoparticles could be derived from the direct fusion of endosomes or the ER/Golgi apparatus axis. On the other hand, both endosomes/lysosomes and secretory vesicles participated in the exocytosis of AuNPs. The IPC analysis further showed that the mitochondria, ER and Golgi apparatus were all involved in transcytosis but not in exocytosis.

### Conventional technologies verified the nano-trafficking mechanism based on proteomics

To verify the nano-trafficking feature based on proteomic analysis and construct an integrated transport mechanism of nanoparticles through the epithelium, conventional technologies, including imageology and pharmacology, were utilized. As illustrated in Figure S9, low temperature significantly reduced the cellular uptake of AuNPs, indicating the particle internalization was energy dependent, and most AuNPs were internalized through the mechanism of endocytosis. It should be noted that a small number of nanoparticles could still enter cells probably by penetrating through the cell membrane even at low temperatures. The IPC analysis also revealed the potential receptor for the endocytosis of AuNPs. The carcinoembryonic antigen-related cell adhesion molecule (CEACAM) was identified as the specific membrane-related protein in the IPC-Trans group (Figure 3B). As one of the pattern recognition receptors (PRRs), CEACAM has been shown to recognize multiple pathogenic bacteria and induce phagocytosis or endocytosis 41. In our study, two isoforms of CEACAM (CEACAM5 and CEACAM6) were found to bind with AuNPs simultaneously and might trigger the subsequent endocytosis of nanoparticles. Further demonstration is still needed to verify this hypothesis.

**Figure 4:**
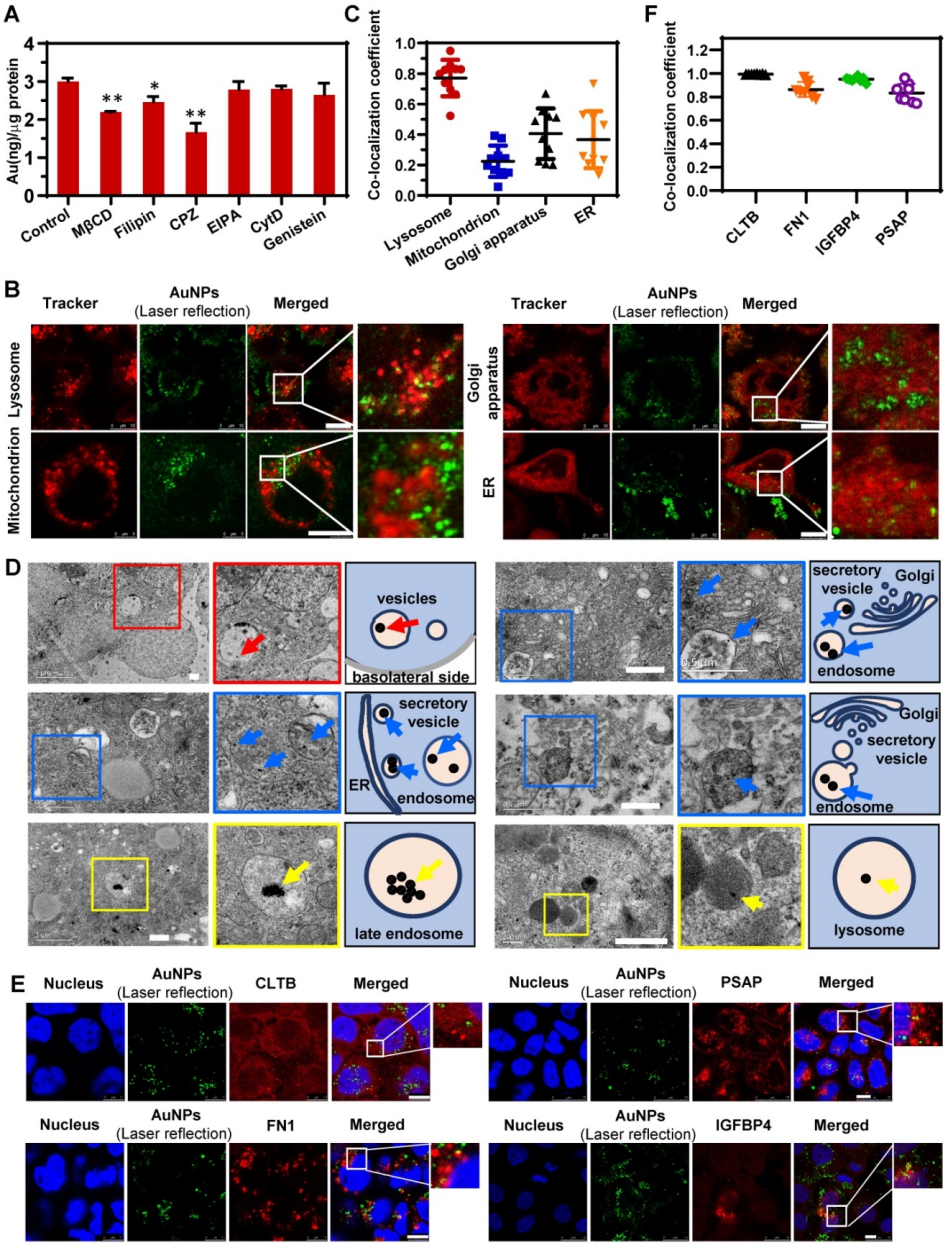
** Nano-trafficking mechanism of AuNPs verified by conventional approaches.** (A) Analysis of the endocytosis mechanism of AuNPs following treatment with different chemical inhibitors. Mean ± SD, n = 3, *p < 0.05 and **p < 0.01. (B) Colocalization images of AuNPs with various organelles (lysosome, endoplasmic reticulum, Golgi apparatus, mitochondrion). Scale bar CLSM, 10 μm. (C) Colocalization coefficients of AuNPs with various organelles (lysosome, endoplasmic reticulum, Golgi apparatus, mitochondrion) indicating percent AuNPs which colocalized with organelles. Mean ± SD, n = 8. (D) Cellular distribution of AuNPs under TEM (left) and its schematic diagram (right). Arrows indicate the AuNPs. Scale bar TEM, 500 nm. (E) Colocalization images of AuNPs with specific IPC-Trans proteins (CLTB, FN1, IGFBP4, and PSAP). Scale bar CLSM, 10 μm. (F) Colocalization coefficients of AuNPs with different IPC-Trans proteins. The colocalization coefficient indicates percent AuNPs which colocalized with IPC-Trans proteins. Mean ± SD, n = 8.

The pharmacological inhibition strategy was used to clarify the detailed pathways during endocytosis. The concentrations of all inhibitors were first optimized to guarantee no cellular cytotoxicity (Figure S10). Compared with the control group (AuNPs without inhibitors), the reagents related to caveolae/lipid raft (CA/LR) and clathrin-mediated endocytosis (CME) exhibited significant inhibition of internalization (Figure 4A). Filipin, a caveolae disrupting reagent, and MβCD, a cholesterol sequestering reagent, were reported to block the endocytosis mediated by caveolae and lipid raft 42. Chlorpromazine (CPZ) was the canonical inhibitor of CME, since it inhibits the assembly of clathrin adapter protein 2 on clathrin-coated pits 43. Ethylisopropylamiloride (EIPA), cytochalasin D, and genistein, all of which reported to be associated with the micropinocytosis 44, 45, did not alter the internalization of AuNPs. These data revealed the involvement of both CA/LR and CME during endocytosis. Interestingly, this conclusion was also consistent with the finding of specific IPC analysis (Figure 3B). Guanine nucleotide-binding protein subunit gamma-12 (GNG12), as one of the specific membrane-related IPC components, is involved in various transmembrane signaling systems and corresponding endocytosis, most of which were associated with the CA/LR pathway 46. Notably, clathrin light chain B (CLTB), a marker protein in CME as one of the components of clathrin coating, was also detected in IPC. In summary, this specific IPC analysis demonstrated the co-existence of CA/LR and CME during transcytosis of AuNPs, corroborating the nano-trafficking mechanism based on traditional pharmacological strategy.

The CLSM images in Figure 4B and the corresponding co-localization analysis in Figure 4C revealed that more AuNPs were transported to late endosomes (LE) and lysosomes compared to mitochondria and ER/Golgi apparatus. Our previous findings, as well as work from other investigators, had shown that the intracellular transport from endosomes to lysosomes contributed little to the transcytosis of nanoparticles 25, 47. Lysosomes might play a role in retaining more nanoparticles in cells, resulting in less transcytosis than endocytosis, which was verified in the current study (Figure 2F). Besides, the IPC analysis identified the binding of only one specific lysosomal protein with nanoparticles (Figure 3B), suggesting a non-critical role of lysosomes in transcytosis. Interestingly, by detecting the colocalization of AuNPs with mitochondria or ER/Golgi apparatus, and identifying the corresponding specific IPC components, it was clear that these organelles were hardly involved in transcytosis. So, there might be other pathway which was dominant for the outgoing transport of AuNPs.

The proteomic analysis of IPC (Figure 3B-D) illustrated that nanoparticles might be internalized by cells into endosomes, and then directly transported to the SV to induce transcytosis. To confirm this, we utilized TEM to detect the subcellular distribution of nanoparticles. As shown in Figure 4D, many AuNP-containing vesicles were located near the basolateral side of cell monolayer (red arrows and insets with red border) and some of them were in the close vicinity of ER or Golgi apparatus, suggesting that these vesicles might be derived from the budding and secretion of ER/Golgi apparatus. Nevertheless, we rarely observed the AuNPs in ER and Golgi apparatus by TEM, indicating that the nanoparticles in secretory vesicles did not originate from the ER/Golgi apparatus. On the contrary, we found a spatial association between AuNP-loaded endosomes and secretory vesicles in TEM images (blue arrows and insets with blue border). This result confirmed the Endo-SV pathway in the transcytosis of AuNPs, verifying the reliability of proteomic-based IPC analysis for the nano-trafficking study. Additionally, other transport pathways of AuNPs were investigated by TEM (Figure 4D). Among different subcellular locations, more nanoparticles gathered in LE and lysosomes (yellow arrows and insets with yellow border), which was consistent with the CLSM result presented in Figure 4B. However, these particles significantly agglomerated to form larger clusters that could not be transported to other organelles causing the intracellular retention. It also explained why transcytosis was lower than endocytosis, as illustrated in Figure 2F.

Next, four different IPC-Trans protein antibodies were used to validate the colocalization of AuNPs with the specific IPC proteins in the Caco-2 monolayer. As shown in Figure 4E, the intracellular AuNPs colocalized with CLTB which was one of the specific IPC proteins located on cell membrane. Secretory proteins, FN1 and IGFBP4, colocalized with AuNPs well. PSAP, a specific IPC lysosomal protein, colocalized with intracellular AuNPs. As shown in Figure 4F, the colocalization coefficients of AuNPs with the four proteins were all above 0.5 indicating good co-localization correlation 48, partially validated the results of specific IPC identification.

In brief, the findings based on the conventional technologies verified and complemented the nano-trafficking features derived from the proteomic identification of IPC. Simultaneously, the comparison revealed the merits of IPC analysis, which is more favorable for the elucidation of molecular mechanisms of nanoparticle transport in cells and generates a large amount of molecular information in limited number of tests.

### IPC as the new nano-bio interface affected post-transcytosis cell transport

**Figure 5:**
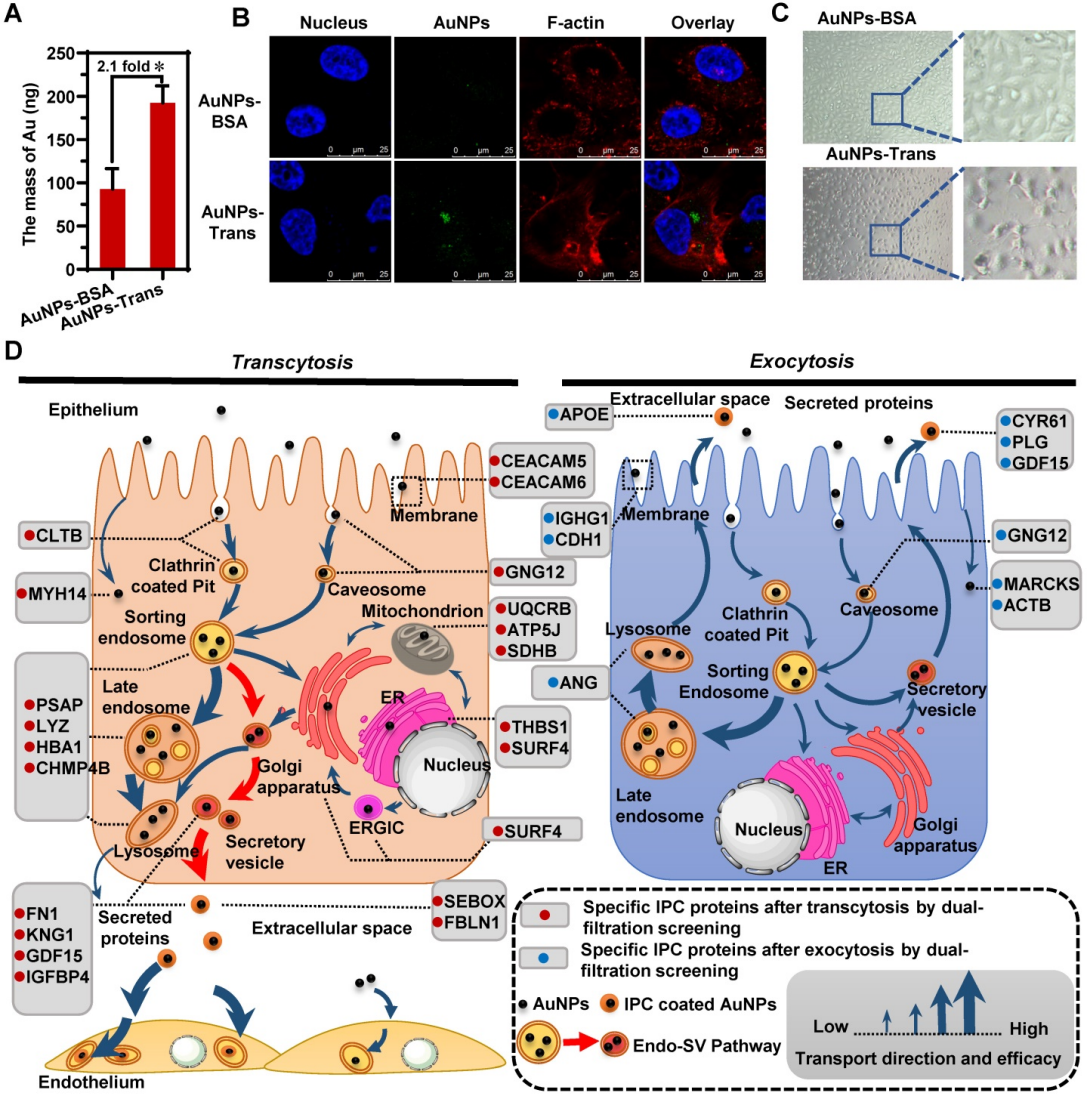
** IPC as the nano-bio interface affected the following cellular transportation after transcytosis.** The intracellular uptake of AuNPs-BSA and AuNPs-Trans by HUVECs evaluated by ICP-MS (A) and CLSM (B). Mean ± SD, n = 3, *p < 0.05. (C) Morphology of HUVECs after incubation with AuNPs-BSA or AuNPs-Trans for 12 h. (D) Schematic diagrams show specific IPC proteins in different cellular transport uncover the preceding and following nano-trafficking features. Based upon the subcellular distribution of specific IPC proteins, a new Endo-SV pathway was found to be the dominant transcytosis route for the nanoparticles (red arrows).

By forming a new interface between nanoparticles and the biological environment, the protein corona mediates the authentic nano-bio interactions 23, 49, 50. Once nanoparticles pass through the epithelium, their transport behavior across the subsequent cell barriers might be affected by the newly formed IPC. In other words, the fate of nanoparticles might be regulated by the surface-bound IPC. To clarify this impact, we cultured human umbilical vein endothelial cells (HUVEC) as the endothelial barrier. The AuNPs were collected in the basolateral chamber after their transcytosis through Caco-2 cell monolayer (AuNPs-Trans) and incubated with HUVECs. The quantitative cellular uptake was detected by inductively coupled plasma mass spectrometry (ICP-MS). As shown in Figure 5A, IPC coating caused internalization of more nanoparticles by HUVECs compared to pristine AuNPs. The CLSM images in Figure 5B also confirmed the same conclusion. These findings demonstrated that the IPC proteins increased the bio-nano interactions. More specifically, specific IPC components might bind to the corresponding receptors in cells to induce increased uptake.

Notably, we identified six extracellular proteins by the IPC analysis (Figure 3B); most of them belonged to the ECM and mediated cell-to-cell and cell-to-matrix interactions. Among them, fibronectin (FN1) is involved in cell adhesion, cell motility and maintenance of cell shape by binding to integrin on the cell surface. Fibulin-1 (FBLN1) plays a role in cell adhesion and migration by incorporating into fibronectin-containing matrix fibers. Thus, the adsorption of these proteins on nanoparticle surface indicated that the ECM-mediated interaction was one of the important triggers for the enhanced cellular uptake of AuNPs. Furthermore, our results also demonstrated that the formation of IPC was beneficial for improving the absorption and transport of nanoparticles through histological barriers.

Importantly, IPC also induced alteration of the endothelium. The phase contrast microscopy images in Figure 5C show that the incubation of AuNPs-Trans caused significant cellular shrinkage with distances between cells broadening compared to the control group (AuNPs-BSA). Consistent with the significant role of mitochondria in cell functions, we presumed that the three specific mitochondrial proteins found in our IPC analysis might be involved in endothelial alterations (Figure 3B). Ubiquinol-cytochrome c reductase complex (UQCRB) is part of the mitochondrial respiratory chain and involved in redox-linked proton pumping. ATP synthase-coupling factor 6 (ATP5J) participates in the production of ATP in the presence of a protein gradient. Succinate dehydrogenase iron-sulfur subunit (SDHB) is also involved in complex II of the mitochondrial electron transport chain and responsible for transferring electrons from succinate to ubiquinone. Generally, all three proteins do not exist in the extracellular space. However, if these mitochondrial proteins are carried out of the organelle as part of the IPC complex, they might trigger the stress response of endothelium. The precise mechanism of this phenomenon remains to be clarified.

## Conclusion

In this study, we confirmed the adsorption of intracellular proteins on particle surface to form protein corona after the transcytosis or exocytosis of nanoparticles through the epithelium. We used a proteomic-based strategy to analyze and identify the IPC components. Our analysis showed that the subcellular properties of IPC proteins could become a connecting link to uncover nano-trafficking features as well as bio-nano interactions. To eliminate the interference of extracellular milieu, a dual-filtration method was established to accurately screen the authentic surface-binding proteins during the cellular transportation of nanoparticles. These “specific” IPC proteins could indeed back-track the transport pathways of nanoparticles in the epithelial cell monolayer. The findings were further verified by conventional imageology and pharmacology techniques, demonstrating the reliability of proteomic-based IPC analysis.

Significantly, by using multiple analytical strategies together with the IPC analysis, we mapped a complete intracellular transport network at a molecular level during the transcytosis and exocytosis of nanoparticles. When nanoparticles transferred to various subcellular structures, they interacted with organelle-specific proteins that became a part of the IPC components. Figure 5D illustrates the atlas of these “marker” IPC proteins in different cellular transportations. Based on the subcellular distribution of IPC proteins and co-localization analysis, we found a new Endo-SV pathway which was dominant for the transcytosis of nanoparticles. It portended that accelerating the conversion from Endo to SV and increasing the SV production would be efficient strategies for improving the transportation of nanomedicines across epithelial barriers.

Besides the back-tracking role, IPC analysis based on proteomics revealed the fate of nanoparticles (Figure 5D). The surface binding of ECM proteins enhanced the nano-bio interactions and increased the uptake of nanoparticles by the subsequent endothelium. However, the existence of some endogenous mitochondrion-related proteins in IPC also induced the cellular stress response. These findings reflected the versatile effects of IPC on cellular behavior.

In summary, the IPC analysis based on a dual-filtration proteomic technology provides an excellent strategy for the nano-trafficking study and lays the foundation for elucidating the potential regulation of transport of nanoparticles at a molecular level.

## Materials and Methods

### Materials

HAuCl_4_•3H_2_O (99%) was obtained from Energy Chemical (Shanghai, China). Sodium citrate was purchased from National Medicine Group Chemical Reagent (Beijing, China). Bovine serum albumin (BSA) was purchased from Amresco (OH, USA). Phosphate-Buffered Saline (PBS) powder, non-essential amino acids, Dulbecco's modified eagle medium (DMEM) and Roswell Park Memorial Institute 1640 containing GlutaMax medium (RPMI 1640) were bought from MACGENE Biotechnology (Beijing, China). Golgi-tracker and Rhodamin-phalloidin were obtained from Yeasen (Shanghai, China). Lyso-tracker, Mito-tracker and ER-tracker were acquired from Thermo Fisher Scientific (Waltham, MA, USA). Rabbit monoclonal to Fibronectin (ab32419, 1:100) was bought from Abcam (Cambridge, UK). CLTB mouse monoclonal antibody (66270-1-Ig, 1:50), IGFBP4 rabbit polyclonal antibody (18500-1-AP, 1:20), and PSAP rabbit polyclonal antibody (10801-1-AP, 1:50) were purchased from Proteintech (Chicago, USA). Donkey anti-rabbit IgG/RBITC and goat anti-mouse IgG/RBITC were bought from Bioss (Beijing, China). Methyl-β-cyclodextrin (MβCD), filipin, chlorpromazine (CPZ), 5-(N-ethyl-N-isopropyl)-amiloride (EIPA) were acquired from Sigma (St. Louis, MO, USA). Cytochalasin (CytD) was obtained from Aladdin (Shanghai, China). Cell lysis buffer for Western blotting and IP and pre-stained color protein ladder were purchased from Beyotime (Shanghai, China). SDS-PAGE gel preparation kit was obtained from Solarbio (Beijing, China).

### Gold nanoparticle preparation and characterization

Gold nanoparticles (AuNPs) were synthesized by reducing hydrogen tetrachloroaurate with sodium citrate as described in our previous work 51. Briefly, 1 mL of 50 mM HAuCl_4_ was added to 49 mL distilled water. The mixture was boiled under reflux, and 5 mL 38.8 mM sodium citrate was added to the mixture quickly. The mixture was stirred for 20 min under boiling condition, and then stirred to room temperature. The obtained AuNPs were dispersed in 0.5 mg/mL BSA aqua at 37 °C and incubated for 30 min to stabilize the nanoparticles. The free BSA was removed by centrifugation (13000 rpm, 30 min, 4 °C) and the pellet of AuNPs was re-suspended in DMEM (without phenol red or fetal bovine serum (FBS)). The stabilized AuNPs were stored at 4 °C for later intracellular transport investigations.

AuNPs with or without BSA coating were mixed with the same volume of phenol red and serum-free DMEM. The appearance of the solutions was photographed after 0 h, 4 h and 4 days. Once the AuNPs were added to DMEM, the solution was dropped onto copper grids and dried. The specimens were imaged by TEM (JEOL, Tokyo, Japan). The diameters of coated AuNPs in the TEM images were measured by Image Pro Plus (IPP) software using manual analysis. The hydrodynamic diameter and zeta potential of AuNPs were measured by the Zetasizer Nano ZS (Malvern Instruments, Worcester, UK) at room temperature. Ultraviolet-visible (UV-Vis) spectrophotometry of AuNPs was performed on UV-VIS Spectrophotometer (Varian, California, USA).

### Cell culture and Cell monolayer construction

Caco-2 and HUVECs were supplied by China Center for Type Culture Collection (Wuhan, China). Caco-2 cells were cultivated in DMEM with 0.1 mM non-essential amino acid, 100 U/mL penicillin, 100 μg/mL streptomycin and 10% FBS. Cells were cultured at 37 °C with 5% CO_2_. After 4 days, the cells were digested with 0.25% (w/v) trypsin, collected, and seeded in a new culture flask or a cell culture plate for the following experiments.

To obtain Caco-2 cell monolayer, Caco-2 cells (2×10^5^) were cultured on the polycarbonate membrane (Transwell, 12-well, 3 μm pore, CORNING). Completed DMEM (0.5 mL) was added to the upper compartment while 1.5 mL was added to the basilar compartment. Medium in both upper and basilar compartments were changed every two days, and the resistance was measured by Volt-Ohm Meter and Accessories (Merck, Darmstadt, Germany) to monitor the integrity of cell monolayer. The monolayer was not used until the TEER was above 500 Ω·cm^2^.

HUVECs were cultured in RPMI 1640 with 100 U/mL penicillin, 100 μg/mL streptomycin and 10% FBS at 37 °C with 5% CO_2_. After 2-3 days, the cells were harvested with 0.25% (w/v) trypsin, collected, and seeded in cell culture plates for the subsequent experiments.

### TEM imaging of the transcytosis of AuNPs on Caco-2 cell monolayer

The integrated Caco-2 cell monolayer was obtained on Transwell membrane. 0.5 mL of 800 μg/mL AuNPs dispersed in DMEM was added to the upper compartment and incubated for 12 h. The Transwell membrane with cell monolayer was cut off and fixed with glutaraldehyde overnight. The longitudinal section of cell monolayer was observed under TEM after a series of sample preparation procedures. The distribution of AuNPs in different parts of the Caco-2 cell monolayer was observed as well as the morphology of tight junctions among Caco-2 cells.

The AuNPs in the basilar compartment and upper compartment of Transwell were collected by centrifugation (13000 rpm, 30 min, 4 °C) separately and re-dispersed in DMEM. AuNPs-BSA and the collected AuNPs were observed under TEM after negatively staining with uranyl acetate solution (1%, w/v).

### Transcytosis efficiency of AuNPs

The concentration of AuNPs and incubation time were chosen as variables to evaluate the transcytosis of AuNPs on Caco-2 monolayer cultured on Transwell porous membrane. To the upper compartment, 0.5 mL of 100 μg/mL, 300 μg/mL or 800 μg/mL AuNPs was added and incubated for 8 h (n = 3). To examine the effect of varying incubation time, 0.5 mL of 500 μg/mL AuNPs was added to the upper compartment and incubated for 6 h, 8 h or 12 h (n = 3). For all experimental groups, AuNPs were collected from the basilar compartments of Transwell by centrifugation (13000 rpm, 30 min, 4 °C). The mass of AuNPs was detected by ICP-MS (Perkin Elmer, Massachusetts, USA).

Also, 0.5 mL of 800 μg/mL AuNPs was added to the upper compartment of Transwell with the Caco-2 cell monolayer and incubated for 12 h (n = 3). Following transcytosis, AuNPs were collected from the basilar compartment of Transwell by centrifugation (12000 rpm, 30 min, 4 °C). Transwell membrane with cell monolayer was cut off and lysed with the cell lysis buffer for 15 min at 4 °C. The lysate was centrifuged (13000 rpm, 30 min, 4 °C) to collect intracellular AuNPs. The mass of AuNPs was measured by ICP-MS.

### Pharmacological and energy inhibition assay of endocytosis

The endocytosis of AuNPs was investigated by the addition of various endocytosis inhibitors as described in Table S1. Caco-2 cells (1×10^5^) were seeded in 12-well culture plates. Before adding AuNPs, the Caco-2 cells were pre-incubated with pharmacological inhibitors at 37 °C for 30 min. Subsequently, 800 μg/mL AuNPs were added to the cells and incubated for 5 h. The concentration of inhibitors was maintained at a constant level. After the incubation, cellular uptake was terminated by adding cold PBS. The cells were washed three times with PBS and lysed with the cell lysis buffer (containing 1 mM PMSF) for 15 min at 4 °C. The concentration of protein was analyzed by the BCA kit, and the mass of AuNPs was detected by ICP-MS. To determine the uptake amount of AuNPs while keeping the number of cells constant, “ng Au/μg protein” was used.

For energy dependence study, 800 μg/mL AuNPs were added to Caco-2 cells in 12-well culture plates and pretreated at 4 °C or 37 °C for 30 min. Subsequently, the culture medium was removed; the corresponding AuNPs were added to the culture plates, and incubated for 6 h at 4 °C or 37 °C. After the incubation, the cells were washed three times with cold PBS and lysed with cell lysis buffer (containing 1 mM PMSF) for 15 min at 4 °C. The concentration of protein was analyzed by the BCA kit, and the mass of AuNPs were detected by ICP-MS. As described previously, “ng Au/μg protein” was used to present the uptake amount of AuNPs keeping the number of cells constant.

### Intracellular distribution analyses of AuNPs

For the co-localization study, four organelle trackers were applied to analyze the colocalization of AuNPs with lysosomes, Golgi apparatus, ER and mitochondria. Caco-2 cells (1×10^5^) were seeded on a confocal dish, and cultured for 5 days to form a cell monolayer. Subsequently, the culture medium was removed. 800 μg/mL AuNPs were added to the confocal dish with Caco-2 cells, and incubated for 6 h. Next, the AuNPs solution was replaced with an organelle tracker solution and incubated for an appropriate time. The co-localization of AuNPs and organelles was detected by CLSM. Laser reflection technology was used here to detect intracellular AuNPs in CLSM (Leica, TCS, SP8, Germany). Most inorganic nanoparticles could be directly detected by detecting the reflected light because of their excellent light scattering characteristics 52. In the CLSM experiment, the excitation laser wavelength was set as 633nm, and the detector was tuned at 620-650 nm to detect the reflected laser signal of AuNPs. Under this condition, the CLSM images of Caco-2 cells incubated with or without AuNPs were captured. The colocalization coefficient of laser reflection signal with organelle fluorescence signal was measured by IPP software. Co-localization coefficient *m_1_*of the Green-Red pixel pair was generated by IPP software, which denoted the percentage of green pixels colocalized with red. The value of *m_1_* above 0.5 indicated colocalization 48.

For TEM observations, 800 μg/mL AuNPs were incubated with Caco-2 monolayer for 12 h at 37 °C. The cells were then collected and fixed with glutaraldehyde overnight. The fixed cell pellet was observed under TEM after a series of sample preparing steps.

### Colocalization analysis of intracellular AuNPs and specific IPC proteins

AuNPs (0.5 mL of 800 μg/mL) were added to the upper compartment of Transwell with Caco-2 cell monolayer and incubated at 37 °C with 5% CO_2_ for 12 h. The Transwell membrane with cell monolayer was cut off, placed on a glass slide, washed with PBS, and fixed with 4% paraformaldehyde for 20 min at room temperature. Subsequently, the monolayer was incubated with 0.2% Triton X-100-PBS for 5 min, and blocked with 5% BSA for 1 h at room temperature. 100 μL diluted specific IPC proteins antibodies (anti-Fibronectin (1:100), anti-CLTB (1:50), anti-IGFBP4 (1:20), and anti-PSAP (1:50)) were added to the monolayer and incubated overnight. After washing 3 times with PBS, 100 μL of fluorescence-labeled secondary antibody (donkey anti-rabbit IgG/RBITC (1:200) or goat anti-mouse IgG/RBITC (1:200)) were added and incubated for 1 h at 37 °C in dark. After labeling cell nuclei with Hoechst 33258, the cell monolayer was preserved with a coverslip and sealed with mountant. Finally, the samples were observed under CLSM (Leica, TCS, SP8, Germany). The colocalization coefficient of laser reflection signal with antibody fluorescence signal was measured by IPP software. Co-localization coefficients *m1* of Green-Red pixel pair was also generated by IPP as above.

### Proteomic identification and analysis of IPC on AuNPs after transcytosis and exocytosis

LC-MS/MS technology based on the label-free quantitative (LFQ) proteomics strategy was utilized to analyze and identify the protein composition in IPC after transcytosis and exocytosis of AuNPs. To identify the specific intracellular proteins, we established a dual-filtration method by excluding the fake IPC proteins that did not interact with nanoparticles during the cellular transportation. (Notably, this method is applicable for studying the nano-trafficking of inorganic nanoparticles in epithelium cells. Inorganic nanoparticles (such as AuNPs, silica nanoparticles and so on) can keep stable during the intracellular transport while organic nanoparticles may degrade. The proteomics analysis on the transcytosis mechanism of organic nanoparticle seems to be much more complicated and remained to be investigated.

To the upper compartments of the Transwell chambers with Caco-2 cell monolayers, 0.5 mL 800 μg/mL AuNPs in cell culture medium or 0.5 mL cell culture medium were added and 1.5 mL cell culture medium was added to the basilar compartments of Transwells in both groups and incubated at 37 °C with 5% CO_2_ for 12 h, here the cell culture medium was serum free. The media in both upper and lower compartments of two groups were collected separately, and cell debris were removed by centrifugation (6000 rpm, 4 °C for 15 min). Meanwhile, the solution in lower and upper compartments of Transwell which was incubated with cell monolayer only were collected separately. And 200 μL 800 μg/mL AuNPs were incubated with the collected solutions or 200 μL Caco-2 cell lysate (containing 1 mg/mL protein) at 37 °C for 30 min as control groups (AuNPs-Baso, AuNPs-Upper and AuNPs-Lysate) (Figure 2G and 3A). AuNPs (AuNPs-Trans, AuNPs-Exo, AuNPs-Baso, AuNPs-Upper and AuNPs-Lysate) were collected by centrifugation (13000 rpm, 4 °C for 30 min) and washed with distilled water 3 times.

For SDS-PAGE, equal mass of collected AuNPs (AuNPs-Trans, AuNPs-Exo, AuNPs-Baso, AuNPs-Upper) were mixed with 20 μL loading buffer and boiled for 5 min at 95 °C. The samples were separated on 10% gel in SDS-PAGE (Bio-Rad, California, USA) at 100 V. The gel was stained with Coomassie brilliant blue and the protein bands on the gels were imaged and quantitative by using the ChemiDoc XRS System (Bio-Rad, California, USA).

For the LC-MS/MS assay, the collected AuNPs were incubated with 40 μL eluant (acetonitrile : trifluoroacetic acid : water = 30% : 3% : 67%) in the oscillator (1000 rpm, 37 °C, 90 min). The mixture was centrifugated (13000 rpm, 4 °C, 30 min), and the supernatant was collected. The precipitate was washed with 20 μL eluant in the oscillator (1000 rpm, 37 °C, 60 min) twice and centrifugated (13000 rpm, 4 °C, 30 min). The supernatants from three elution steps were mixed, and the concentration of proteins was measured by Bradford assay.

The protein digestion was executed using tube-gel method as described in our previous work 53. 3 μL protein sample (3 μg protein for each group), 4.2 μL of pH 8.8 Tris-HCl buffer, 2.5 μL of 30% acrylamide solution, 0.1 μL 10% SDS and 1.0 μL of 10% ammonium persulfate were added to a 1.5 mL tube and mixed, and 0.1 μL TEMED was added to prepare tube-gel. Then the gel was fixed with 50% methanol, 12% acetic acid for 30 min and chopped into pieces. After dehydration with acetonitrile (ACN), reduction with 10 mM TCEP, and alkylation with 50 mM iodoacetamide, the gel pieces were washed with 50% CAN/50 mM NH_4_HCO_3_ buffer and dehydrated with ACN twice. The gel was rehydrated with 10 μL of 10 ng/μL trypsin in 25 mM NH_4_HCO_3_ buffer at 4 °C for 2 h and then 37 °C overnight. After that, the peptide mixture was extracted twice with 5% FA / ACN (1:2) and lyophilized.

The peptides (10 μL, 1 μg) were loaded on a C18 pre-column (No. SC100, Thermo Scientific, MA, USA) and separated on the analytical column (C18-A2, 75 μm × 10 cm, No. SC 200, Thermo Scientific, MA, USA) using an Easy-LC nano-HPLC (Thermo Scientific, MA, USA). For the gradient separation, the mobile phase A (aqueous solution containing 0.1% FA (Fisher Scientific)) and the mobile phase B (acetonitrile ((Fisher Chemical, HPLC grade) solution containing 0.1% FA) were set as 5%~ 30% for 65 min, 30%~50% for 10 min, 50%~100% for 10 min, and held at 100% for 5 min. Meanwhile, the flow rate kept at 0.3 μL / min. LTQ Orbitrap Velos pro (Thermo Scientific, MA, USA) was used for mass spectrometric analysis. The spray voltage was set at 2.5 kV with the ion transfer capillary at 250 °C. The MS/MS spectra were acquired in a data-dependent collision induced dissociation (CID) mode, and the full MS was obtained from 350 to 2000 m/z with resolution 60, 000. The top 15 most intense ions were selected for MS/MS analysis. Parameters for acquiring CID were set as follows, activation time: 10 ms, Q-activation: 0.25, normalized energy: 35. The dynamic exclusion was as follows, duration: 30 s, repeat count: 1, exclusion list size: 500, exclusion duration: 60 s.

Raw files were analyzed using Proteome Discoverer v1.4.1.14 (Thermo Scientific, MA, USA). MS/MS spectra were searched against UniProt Human database (Sep 2014, 146,661 entries). The search parameters were as follows, variable modification of methionine oxidation: +15.995 Da, fixed modification of cysteine residues: +57.021 Da, full trypsin cleavage, at most one missed tryptic cleavage site, error tolerance in MS: 10 ppm and error tolerance in MS/MS: 0.8 Da. False discovery rates were acquired by Percolator selecting identification with a q-value equal or less than 0.01. Peptide spectral matches were further filtered based on the selected peptide confident with high.

### Investigation of intracellular uptake of AuNPs-Trans by the endothelium

HUVECs (4×10^4^ /mL) were seeded in 96-well plates and cultured for 24 h. Then, the cells were incubated with 36 μg/mL AuNPs-Trans or AuNPs-BSA for 12 h. The morphology of HUVECs was observed by an optical microscope. The cells were washed three times with PBS and lysed with cell lysis buffer for 15 min at 4 °C. The mass of AuNPs was detected by ICP-MS.

AuNPs-Trans or AuNPs-BSA, 36 μg/mL each, were incubated with Caco-2 cells seeded in confocal dishes for 12 h. The cells were washed three times with cold PBS, fixed with 4% paraformaldehyde, punched with 0.2% Triton X-100-PBS for 5 min, and incubated with Rhodamine-phalloidin (dispersed in 1% BSA TPBS solution) for 30 min at 37 °C. Subsequently, the cells were incubated with 5 μg/mL Hoechst 33258 at 37 °C for another 30 min. Finally, the intracellular uptake of AuNPs was observed under CLSM (Leica, TCS, SP8, Germany).

### Statistical data analysis

Quantification data shown as mean ± standard deviation (SD) were obtained at least by three independent experiments. The data was analyzed by using GraphPad Prism software. *p*-value was acquired by two-tailed unpaired Student's t-test for *p < 0.05, **p < 0.01, ***p < 0.001 and ****p < 0.0001.

### Data availability

The mass spectrometry proteomics data have been deposited to the ProteomeXchange Consortium via the PRIDE 54 partner repository with the dataset identifier PXD015651.

## Supplementary Material

Supplementary figures and table.

Supplementary LFQ Proteimics identification.

## Abbreviations

AuNPs: gold nanoparticles
BSA: bovine serum albumin
CA/LR: caveolae/lipid raft
CC: cellular component
CLSM: confocal laser scanning microscopy
CME: clathrin-mediated endocytosis
CPZ: Chlorpromazine
DLS: dynamic light scattering
ECM: extracellular matrix
ER: endoplasmic reticulum
ERGIC: the ER-Golgi intermediate compartment
Endo-SV: endosomes to secretory vesicles
ES: endosomes
GO: gene ontology
HUVEC: human umbilical vein endothelial cells
ICP-MS: inductively coupled plasma mass spectrometry
IPC: intracellular protein corona
IPP: Image pro plus software
LC-MS/MS: Liquid chromatography tandem mass spectrometry
LE: late endosomes
LFQ: label-free quantitative
MW: molecular weight
MS: mass spectrometry
PC: protein corona
PRRs: pattern recognition receptors
SDS-PAGE: sodium dodecyl sulfate polyacrylamide gel electrophoresis
SV: secretory vesicle
TEER: trans-epithelial electric resistance
TEM: transmission electron microscopy
