# Supplementary material for: Proteomic analysis of intracellular protein corona of nanoparticles elucidates nano-trafficking network and nano-bio interactions
Source: Theranostics. 2020 Jan 1;10(3):1213–29. doi: 10.7150/thno.38900 (PMC6956802; doi:10.7150/thno.38900)
Supplement: Supplementary file 1 — Supplementary figures and table. [file thnov10p1213s1.pdf]

Supplementary figures and table

Proteomic analysis of intracellular protein corona of nanoparticles elucidates nano-trafficking network and nano-bio interactions

Mengmeng Qin[1, 2], Jian Zhang[1, 2], Minghui Li[1, 2], Dan Yang[3], Dechun Liu[1, 2], Siyang Song[1, 2], Jijun Fu[4], Hua Zhang[1, 2], Wenbing Dai[1, 2], Xueqing Wang[1, 2], Yiguang Wang[1, 2], Bing He[1, 2, *] and Qiang Zhang[1, 2, *]

1. Beijing Key Laboratory of Molecular Pharmaceutics and New Drug Delivery Systems, School of Pharmaceutical Sciences, Peking University, Beijing, 100191, China.

2. State Key Laboratory of Natural and Biomimetic Drugs, Peking University, Beijing, 100191, China.

3. School of Food and Biological Engineering, Shaanxi University of Science and Technology, Xi'an, 710021, China.

4. School of Pharmaceutical Science, Guangzhou Medical University, Guangzhou, 511436, China.

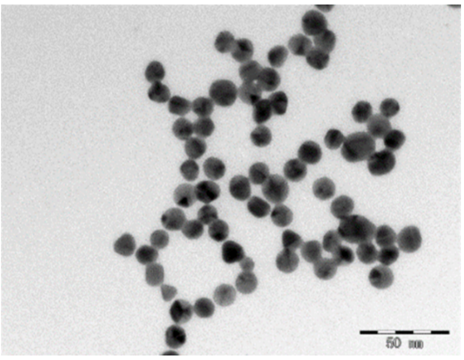

Figure S1. Morphology of newly synthesized gold nanoparticles (AuNPs) with average diameter of 12 nm under TEM.

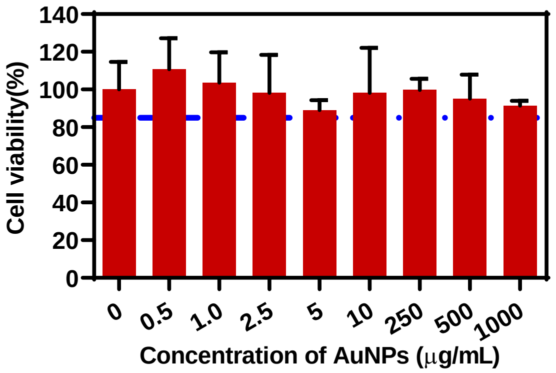

Figure S2. Cell viability of Caco-2 cells under different concentration of AuNPs for 12 h measured by MTT method (n = 6). The cell viability of each group is above 85%.

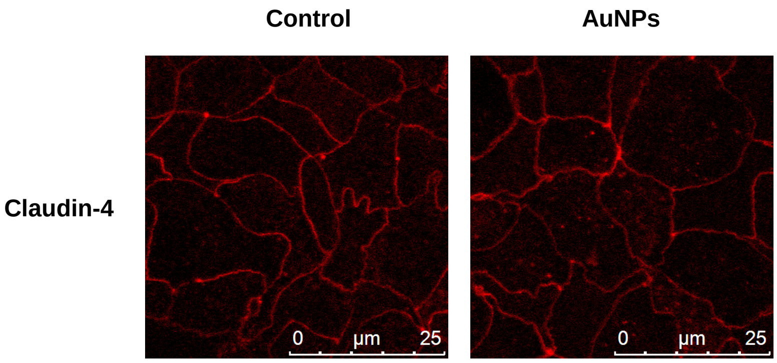

Figure S3. Continuous distribution of claudin-4 in the cell borders of Caco-2 monolayer incubated with or without AuNPs for 12 h.

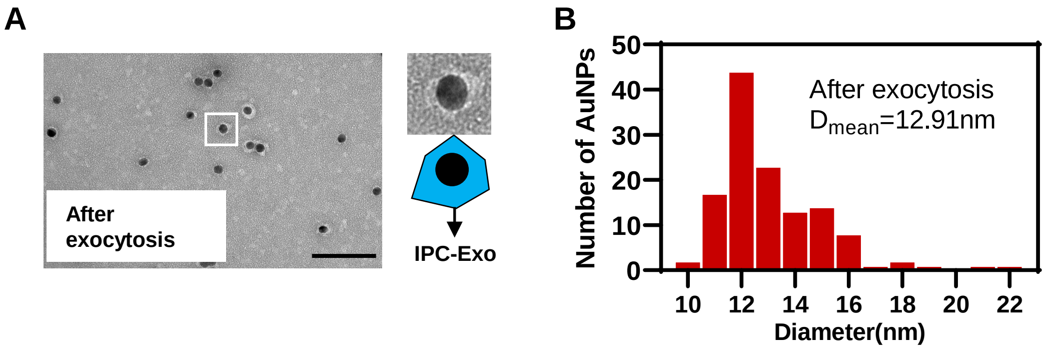

Figure S4. (A) Morphology of AuNPs after exocytosis captured by negative stained TEM. Scale bar TEM, 100 nm. (B) Diameter distribution of AuNPs after exocytosis. The diameter of AuNPs was measured by IPP software according to the TEM photos. n > 200.

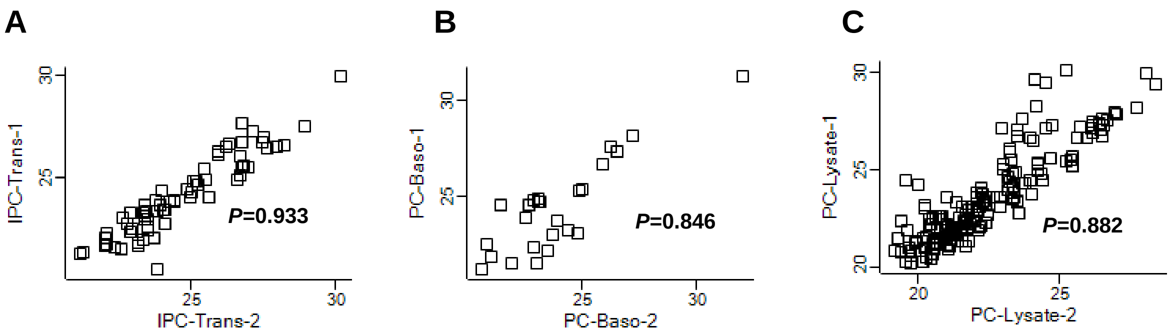

Figure S5. Correlation analysis of the label-free quantitative data from two independent biological replicates for IPC-Trans (A), PC-Baso (B) and PC-Lysate (C) groups performed by Perseus (a proteomic data analytical software). All Pearson correlation coefficient values exceeded 0.8, indicating the LFQ proteomics was feasible for the protein corona analysis in this study.

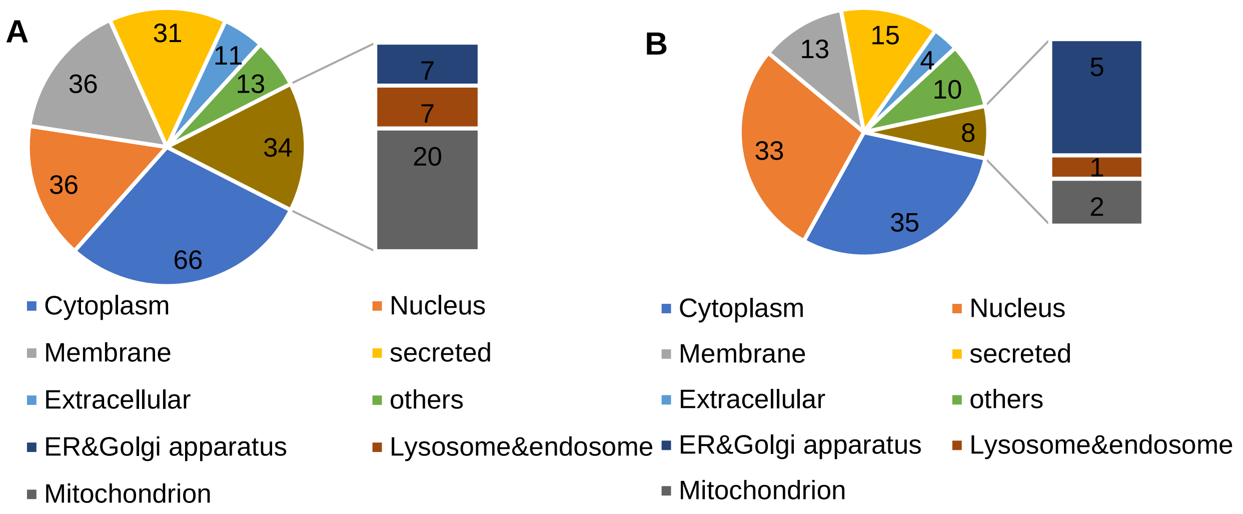

Figure S6. Statistics of subcellular distribution of proteins in IPC-Trans (A) and IPC-Exo (B).

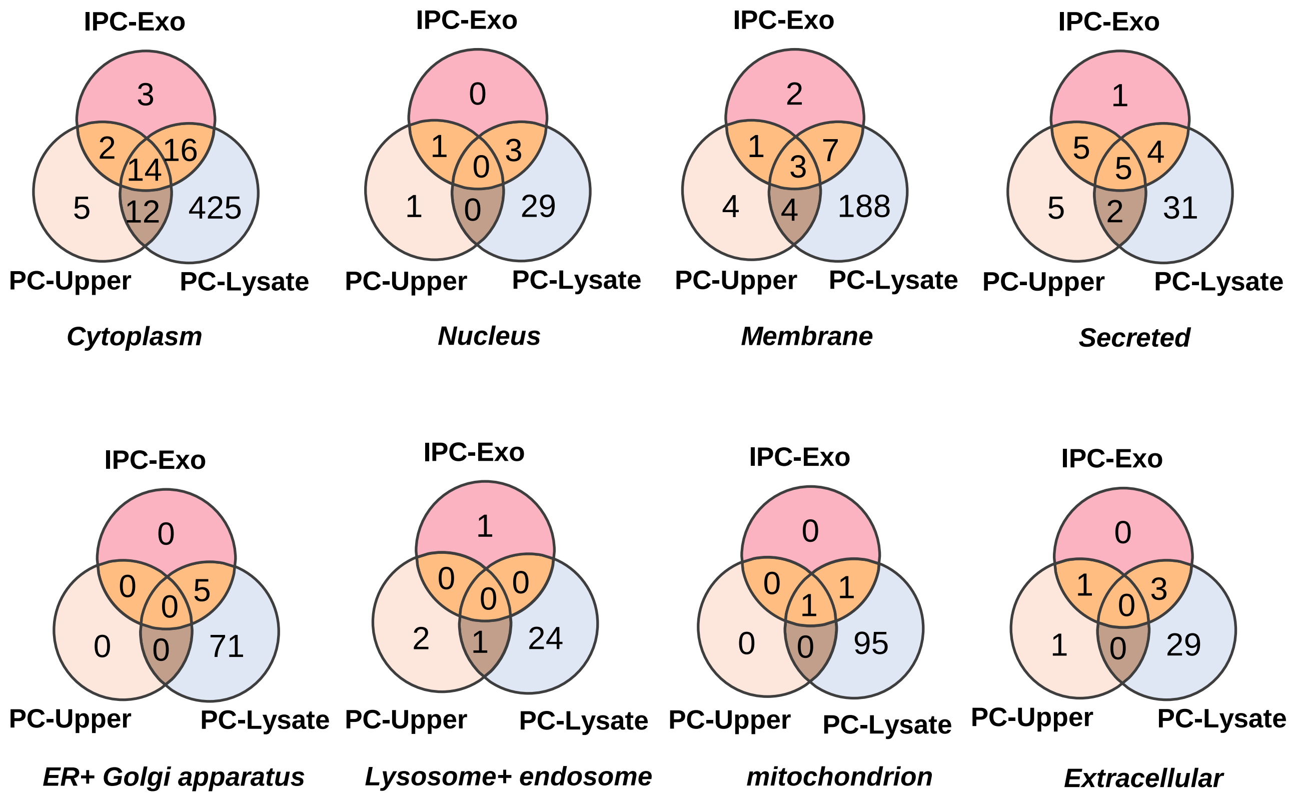

Figure S7. Venn diagrams of identified proteins among different PC groups. The pink area indicated the specific IPC and the yellow area referred to the shared IPC proteins of IPC-Exo over PC-Upper or PC-Lysate.

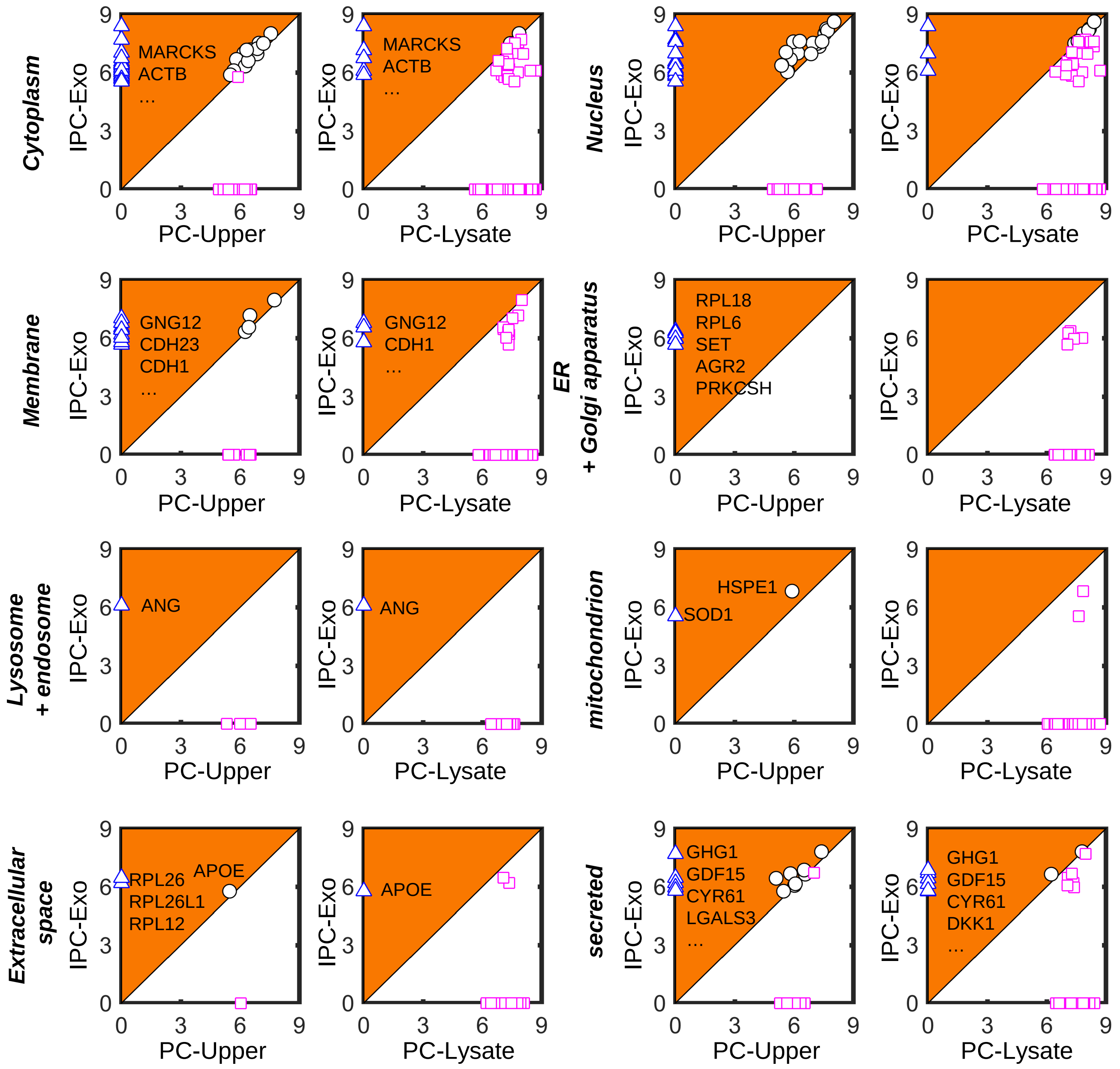

Figure S8. Comparation of LFQ intensities (log10 LFQ) of proteins located in cytoplasm, nucleus, membrane, ER and Golgi apparatus, lysosome and endosome, mitochondrion, extracellular space and secreted between IPC-Exo and PC-Upper or between IPC-Exo and PC-Lysate. The data point above the diagonal line indicated the higher surface adsorption in IPC-Exo group compared to the other two reference groups for the same protein.

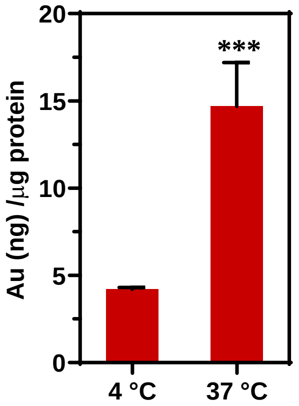

Figure S9. Energy-dependence of AuNPs in endocytosis was evaluated through low-temperature incubation. Mean ± SD, n = 3, ***$p < 0.001$.

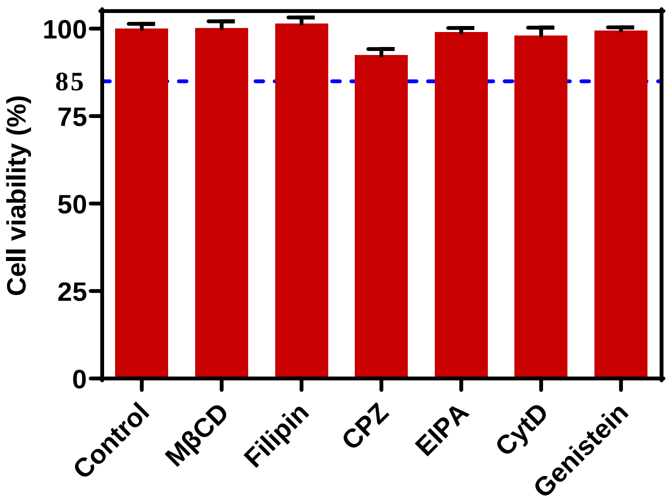

Figure S10. Cell viability of Caco-2 cells under different endocytosis inhibitors, the concentration of inhibitors was listed in Table S1.

Table S1. Concentration of inhibitors used for endocytosis study.

Inhibitors	MβCD	Filipin	CPZ	EIPA	CytD	Genistein
Concentration	10 µM	2.5 µg/mL	50 µM	100 µM	0.5 µM	100 µM
